# Non-Neuronal Acetylcholine: The Missing Link Between Sepsis, Cancer, and Delirium?

**DOI:** 10.3389/fmed.2015.00056

**Published:** 2015-08-21

**Authors:** Adonis Sfera, Michael Cummings, Carolina Osorio

**Affiliations:** ^1^Psychiatry, Patton State Hospital, Patton, CA, USA; ^2^Psychiatry, Loma Linda University, Loma Linda, CA, USA

**Keywords:** acetylcholine, cell cycle, inflammation, carcinogenesis, immunosuppression

## Abstract

The interaction between living organisms and the environment requires a balancing act between genomic and epigenomic forces. Inflammation and cellular proliferation are kept in check by the genes, which code for their components and the microRNAs, which are capable of silencing the transcription of these genes. Acetylcholine (ACh) may play a unique role in the maintenance of this equilibrium, as the epigenomic inhibition of the gene coding for nicotinic receptors, and disinhibits the gene causing anergia in immune cells. We hypothesize that age-induced ACh deficiency is the result of an epigenomic dysfunction of microRNA-6775 (miR-6775), which silences the transcription of CHRNA7 gene [coding for alpha 7 nicotinic cholinergic receptors (nAChRs)]. When silenced, this gene induces decreased expression of alpha 7 nAChRs, which may predispose elderly individuals to inflammation, neuroinflammation, and delirium. We hypothesize further that miR-6775-induced hypocholinergia augments the expression of RNF 128, the gene related to anergy in lymphocytes (GRAIL). This gene favors regulatory T cells (Tregs), promoters of immunologic tolerance, which may predispose to both cancer and sepsis-induced immunosuppression.

## Introduction

Altered immunity, low-grade inflammation, and neuroinflammation accompany old age and may predispose to infections often progressing to sepsis, sepsis-induced delirium (SID), and cancer. Recent studies have shown that aging is associated with a body-wide decrease in nicotine binding to nicotinic acetylcholine receptors (nAChRs) (1, 2). Acetylcholine (ACh) depletion is one of the best-documented hypotheses of delirium, and it is a common knowledge that old age and anticholinergic medications predispose to delirium. Recently, it was demonstrated that a preexisting impairment in cholinergic signaling was necessary for the pathogenesis of delirium (3).

Non-neuronal ACh is known to participate in cellular proliferation, differentiation, and apoptosis throughout the body tissues and organs; however, a direct link between ACh and immune cells’ anergia has not been demonstrated. In this article, we hypothesize that age-related hypocholinergia predisposes not only to delirium but also to immunosenescence, which is often associated with increased risk for cancer and sepsis-induced immunosuppression (SAIS).

## Method

In order to test this hypothesis, we conducted a simple public domain search utilizing available on-line microRNA tools, including EXIQON miRSearch, DIANA-miRPath v3.0, and Wizemann Institute of Science miRBase, and identified miR-6775 (accession number MIMAT0027451) as being a regulator of both RNF 128 and CHRNA7 genes.

## Aging, ACh, and Low-Grade Inflammation

The immune competent cells of the human body must be aggressive enough to “fire” promptly at intruding pathogens, but tolerant enough to avoid autoimmune “friendly fire.” This delicate balance between too much and not enough inflammation is facilitated by a tight cooperation between the genom and the epigenom. Lack of epigenomic supervision, such as microRNA dysfunctions, may manifest as impairment in immunity or autoimmunity both of which are prevalent in elderly.

**Figure 1:**
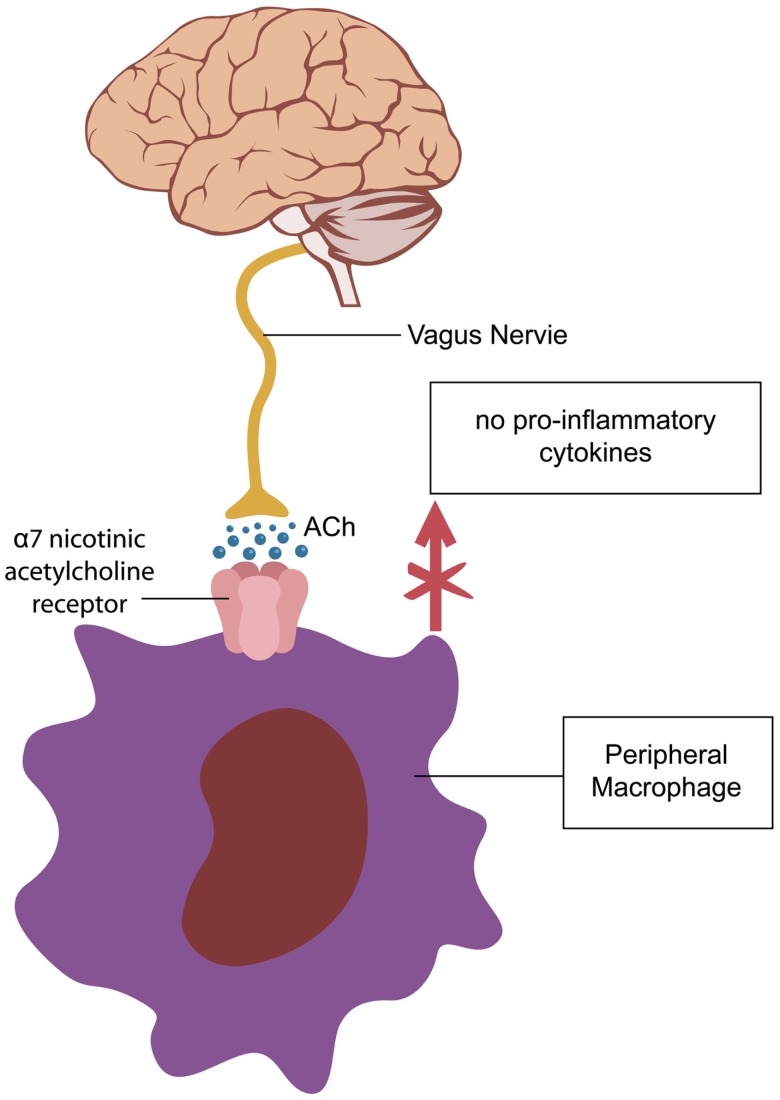
**Activation of alpha 7-cholinergic nicotinic receptors (nAChR) on peripheral macrophages decreases cytokine synthesis exerting an anti-inflammatory effect (5)**. A similar cholinergic pathway, regulating microglial activation is operational in the CNS (6, 7).

Recent studies demonstrate that aging is associated with lowered transcription of nAChRs with subsequent low-grade inflammation, as failure to properly activate alpha 7 nAChRs results in release of pro-inflammatory cytokines (Figure 1). For example, postmortem studies of human brains with Alzheimer’s disease (AD) reported up to 70% decrease in nAChRs expression (2). In addition, it was demonstrated that beta-amyloid itself can decrease transcription of nAChRs. With the same token, in sepsis, low expression of alpha 7 nAChRs on peripheral blood mononuclear cells is associated with uncontrolled inflammation and poor prognosis, suggesting age-related predisposition for sepsis (4). Furthermore, recent studies show that nicotine protects against septic injury by activation of alpha 7 nAChRs, which probably inhibits tall-like receptors 4 (TLR4) (5).

Studies of prostaglandin pathways, such as cyclooxygenase (COX)-1 in microglia and perivascular macrophages, demonstrate that alteration in these pathways may predispose to cognitive dysfunction. Subsequently, COX-1 inhibitor SC-560 provides significant protection against LPS-induced cognitive deficits (8). These studies are consistent with the neuroinflammatory model of delirium. In addition, preclinical studies showed that COX-1 deficient (COX-1^−/−^) mice show not only reduced neuroinflammation and neuronal damage but also decreased cellular proliferation after lipopolysaccharide injection (9). Furthermore, during infection and inflammation, COXs enhance generation of prostaglandin E2 (PGE2). PGE2 is an immune-stimulator as it promotes T-cell activation and proliferation. Therefore, a dysfunction of miR-6775, silencing the expression of RNF 128 gene may result in excessive immune cells activation, including microglia via PGE2. This pathway may help explain the beneficial effect of COX-1 inhibitors in cognitive dysfunctions (10).

The RNF 128 gene codes for an enzyme, an ubiquitin ligase, which inhibits the transcription of IL-2 and IL-4 cytokine genes. Silencing these genes triggers proliferation of Tregs, the anergic T cell phenotype (11, 12). Tregs were shown to decrease mitogen-induced lymphocyte proliferation by directing these cells into apoptotic pathways, impairing immune responses (13, 14). Furthermore, by promoting immune tolerance, Tregs are utilized in post-transplant management as they suppress allograft rejection (15). On the other hand, the absence of Tregs was demonstrated to trigger autoimmunity and inflammation by disinhibition of pro-inflammatory T helper cells 17 (Th17) (16). These cells access the CNS via diapedesis or through the recently described dural lymphatic vessels, spreading the inflammation into the brain (17). Interestingly, the proliferation of Th17 is augmented by the absence of alpha 7 nAChRs, suggesting that silencing CHRNA 7 gene, probably by microRNA, induces a Th17 response (16).

MicroRNAs (miRs) are a family of small non-coding RNAs, which are physiologically able to silence the expression of genes, which in return may activate or inhibit next tier genes. Therefore, a dysfunctional miR may decrease or increase transcription of genes depending on whether it silences the activator or the inhibitor of a particular gene. For example, aging was demonstrated to increase miR-23b, but decrease miR-17, miR-19b, miR-20a, and mir-106a, probably by silencing the activator genes (18, 19). Interestingly, miR-23b was demonstrated to safeguard against autoimmunity and to regulate endothelial cells inflammation in sepsis (20, 21).

## Non-Neuronal ACh and the Cell Cycle

It has been known for more than a decade that peripheral lymphocytes derived from patients with AD proliferate less to mitogenic stimulation than lymphocytes derived from normal individuals, but a connection between the immune cells’ proliferation, apoptosis, and ACh signaling has not been established (22, 23). ACh is well known for its neurotransmitter role at the synapses and the neuromuscular junctions, but its non-neuronal functions have only been documented lately (24). Non-neuronal ACh is released by different cells into the extracellular space (ECS) of numerous tissues, including the brain. Here, it operates in paracrine fashion, mainly by modulating cellular cytoskeleton, adapting it for proliferation, differentiation, or apoptosis, depending on the immediate cellular need (24). These non-neurotransmitter functions of ACh were documented both at phylogenetic and ontogenetic levels (25, 26). For example, phylogenetically, ACh was present in prokaryotic, eukaryotic cells, protozoa, fungi, and plants long before the evolution of a nervous system; ontogenetically, ACh has been detected in human embryos prior to neurogenesis or synaptogenesis (27).

The cholinergic system described on peripheral lymphocytes consists of choline acetyltransferase (ChAT), muscarinic and nicotinic ACh receptors (mAChRs and nAChRs), and acetylcholinesterase (AChE). Aside from inflammation, this system plays a major role in lymphocyte proliferation on antigen contact (26, 27). A non-neuronal cholinergic system was also described in the CNS comprised astrocytes, microglia, and endothelial cells (27). It was recently demonstrated that astrocytes secrete ChAT and that both neuronal and non-neuronal cells express nicotinic and muscarinic receptors, suggesting that inflammation and cellular proliferation may utilize paracrine ACh signaling (28). In the CNS, non-neuronal ACh is synthesized in the ECS from choline and acetyl coenzyme A in a reaction catalyzed by the astrocyte-secreted ChAT (28). Astrocytic pathology may impair ChAT biosynthesis, predisposing to inflammation and aberrant cellular proliferation (29). For example, deficient ACh signaling may induce post-mitotic neurons to enter the cell cycle, as demonstrated in AD. In this condition, neurons undergo apoptosis as they lack the cellular machinery to complete mitosis; they enter the cell cycle, progress through the S phase, arrest in the G2 phase, and undergo pre-programed cell death (30–32).

Aside from affecting the neurons, aging was demonstrated to induce similar changes in astrocytes and microglia. For example, preclinical studies demonstrate that aging facilitates upregulation of astrocytic aquaporin-4 (AQP-4) proteins with resultant cytotoxic edema. Edematous astrocytes may be unable to synthesize ChAT, further impairing cholinergic signaling, and predisposing to delirium (33, 34). AQP-4 upregulation and astrocytic swelling were demonstrated in a variety of age-related diseases, including SID, stroke, traumatic brain injury (TBI), brain tumors or metastases, brain abscesses, and dehydration (34).

Astrocytic cytotoxic edema with decreased ACh signaling may be responsible for the aberrant neuronal re-entry into the cell cycle (34). Interestingly, another microRNA, miR-6739 (accession number MIMAT0027379), controls three genes: the cell division and apoptosis regulator 1 (CCAR1, ENSG 00000060339), regulator of cell cycle (RGCC, ENSG00000102760), and Meteorin glial cell differentiation regulator (METRN, ENSG 00000103260). These genes may link age-related changes in immunity with astrocyte pathology and neuronal re-entry into the cell cycle. Moreover, miR-6775 and miR-6739 may regulate central and peripheral immunity (astrocytes are part of the innate immune system) and keep neuronal cell cycle in check, assuring stability of these cells in G0 phase.

Microglia are also components of the innate immune system, and impairments in cholinergic signaling were described in aging microglia. Interestingly, preclinical studies show that selective removal of senescent microglia from the brain of older animals can delay or prevent neurodegeneration (35). In addition, microglia-mediated neuroinflammation via TLR4 was hypothesized in the pathogenesis of post-operative delirium (36). Studies in rodents demonstrate that CHRNA 7 gene-induced expression of alpha 7 nAChRs suppresses microglial inflammation and shifts these cells toward restorative phenotypes (37, 38). Therefore, miR-6775-induced silencing of CHRNA 7 gene favors microglial pro-inflammatory phenotype.

Other post-mitotic cells, such as cardiomyocytes, are known for their ability to re-enter the cell cycle and to synthesize ACh (39, 40). Recent studies demonstrate that ACh deficiency may induce apoptosis in cardiomyocytes, while vagal electrical stimulation in rodents was found to protect against it (41). In addition, the role of ACh in inducing cell cycle entry is relevant for carcinogenesis. For example, mAChRs activation in small cell lung carcinoma induces cancer cells to arrest in S and G2/M phases with a concomitant decrease in DNA synthesis (42). In human glioblastoma cells, ACh inhibits malignant cells’ entry into the cell cycle via M2 receptor activation (43). Recently, it was demonstrated that cholinesterase enzymes decrease malignant cells’ proliferation in hepatocellular carcinoma (44). In other cancers, ACh seems to promote malignant growth, probably depending on the subtype of muscarinic receptors activated. For example, in colon cancer, upregulation of M3 muscarinic receptors is associated with increased malignant cells’ proliferation (45).

Taken together these data suggest that ACh binding to mAChRs, expressed by lymphocytes, triggers their proliferation in response to mitogens. Conversely, lack of such binding, caused by deficient ACh, may result in immune anergia (12). On the other hand, in post-mitotic neurons, binding of ACh to mAChRs (or receptor subtypes) may keep the cell cycle in check by preventing exit from G0 phase. Deficient ACh binding may induce cell cycle instability with aberrant attempts to initiate mitosis (46).

## Conclusion

The human immune system is hard-wired with the CNS by autonomic fibers and by the newly described dural lymphatic vessels. Low grade inflammation and neurosenescence were described in the elderly both in the immune system and the CNS, predisposing to aging pathology, such as infections, sepsis, and delirium. Dysfunctional miRs may be responsible for the age-related impairment in cholinergic signaling, inducing both inflammation and neuronal apoptosis after aberrant attempts to initiate the cell cycle. miR-6775 and miR-6739 may modulate inflammation, cell cycle, apoptosis, and glial functions, and may qualify for the name of “deliromiRs.”

## Conflict of Interest Statement

The authors declare that the research was conducted in the absence of any commercial or financial relationships that could be construed as a potential conflict of interest.
